# Ligation of lymph vessels for the treatment of recurrent inguinal lymphoceles following lymphadenectomy

**DOI:** 10.1186/s12957-016-0766-z

**Published:** 2016-01-13

**Authors:** Navid Mohamadpour Toyserkani, Henrik Toft Nielsen, Vivi Bakholdt, Jens Ahm Sørensen

**Affiliations:** Department of Plastic and Reconstructive Surgery, Odense University Hospital, Sdr. Boulevard 29, DK-5000 Odense C, Denmark

## Abstract

**Background:**

Recurrent lymphocele following groin dissection is generally a self-limiting condition, but in a few cases, the lymphocele persists and for this, there are not many options. Few reports have proposed the efficacy of lymph vessel ligation with patent blue as a vessel locator. We have used this technique since 2007 in our very severe cases and herein present our results.

**Methods:**

The study was a retrospective case series in a university hospital setting. All patients who had this procedure performed were included from the first procedure performed in 2007 until August 2015, and their data was retrieved from electronic patient records.

**Results:**

In total, eight patients had this procedure performed for a total of ten inguinal regions. In all regions, leaking lymph vessels were easily found by the blue color and a median of 3 (range 1–5 vessels) vessels per region were ligated using titanium clips. For two patients, there was still a need for puncture which lasted 13–37 days postoperatively. For the remaining patients, there was an immediate stop in lymphocele formation but one patient developed a lymphatic malformation which after removal resulted in the recurrence of lymphocele and had the procedure performed again with immediate effect.

**Conclusions:**

Ligation of lymph vessels for the treatment of recurrent inguinal lymphoceles appears to be an appropriate treatment modality that is both quick and easy to perform with minimum risk, and in most cases, it results in immediate complete stop in the lymphocele formation.

## Background

Inguinal lymphadenectomy is depending on cancer stage, a standard part of surgical treatment of several cancer types (squamous cell carcinoma, melanoma, etc). This treatment is associated with high rates of morbidity which include wound infection, necrosis of skin flaps, lymphedema, and also recurrent lymphoceles in the surgical site [1].

Recurrent lymphoceles may be without symptoms but can also be associated with pain and movement restriction. The occurrence of lymphoceles after inguinal lymphadenectomy is very common with an incidence of 11 % in melanoma patients [2], and similar numbers are produced for other cancer types.

Many different approaches have been tried to prevent or minimize lymphocele formation with inconsistent results. In general, this condition is self-limiting, but in some cases, there is no end in sight for the recurrence of the lymphocele. In the last decades, a few case reports and small series have suggested that using patent blue to identify leaking lymph vessels could be the solution to this problem [3–5].

Since 2008, we have used this technique in cases where the lymphocele formation was either unmanageable or where there was no end in sight for the lymphocele formation. We present our results with the technique.

## Methods

This study was conducted as a retrospective study in a tertiary hospital department setting. All patients that had previously had lymph vessel ligature were identified through our electronic patient records using relevant surgical procedure codes. In addition, operating theater lists were checked manually to ensure complete inclusion. The inclusion dates ranged from 2004 (January) to 2015(August). Case files were read, and demographic, procedure, and outcome-related data was gathered.

All patients were treated in compliance with the Helsinki Declaration. Patients included in this retrospective study were not treated as part of a clinical study. Ethical approval from an ethics committee was not needed for this retrospective study.

**Fig. 1:**
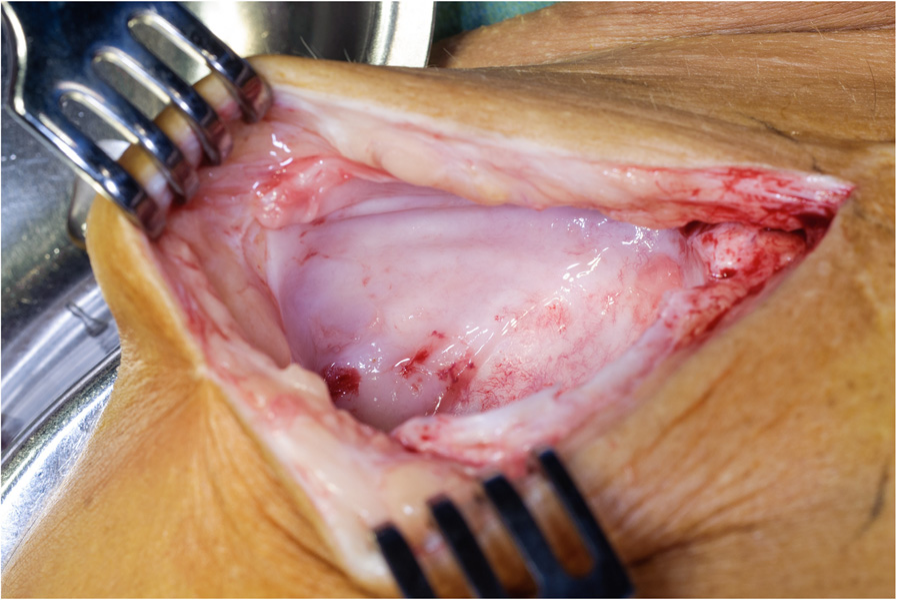
Intraoperative photograph showing the cavity before patent blue injection

**Fig. 2:**
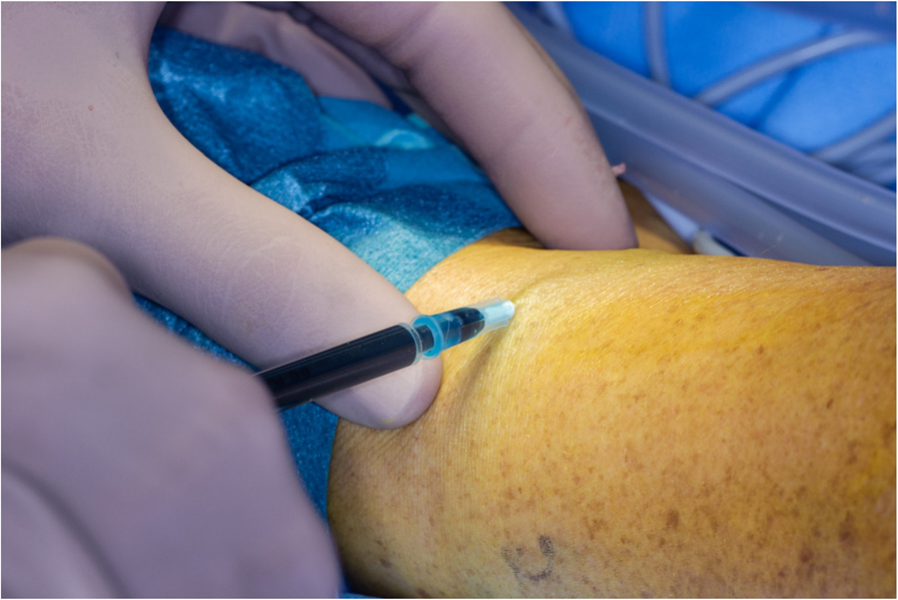
Intraoperative photograph showing injection of patent blue at the level of the ankle

**Fig. 3:**
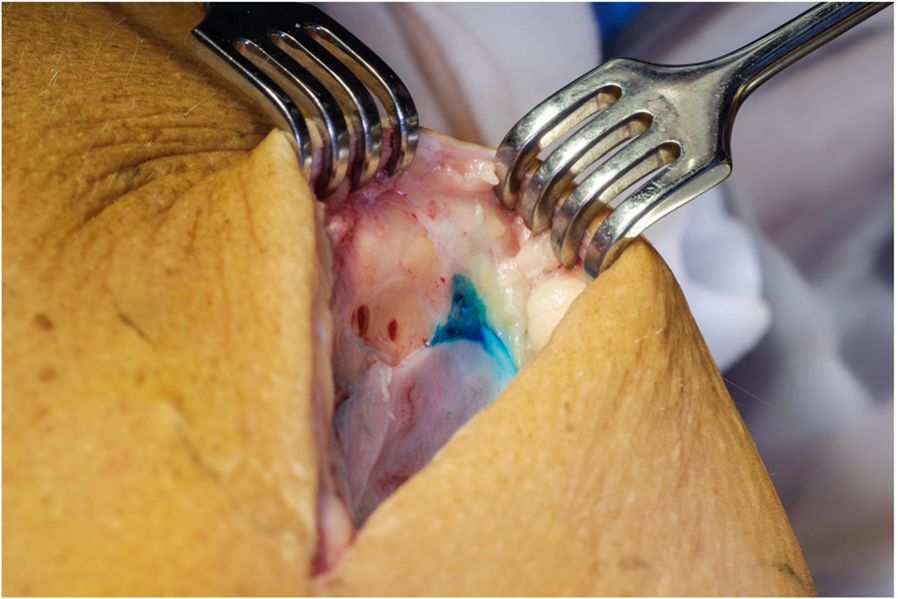
Intraoperative photograph showing the blue leaking lymph vessel in the cavity five minutes after injection of patent blue

The surgical procedure began in all cases with the injection of patent blue at the level of the ankle with the injection of 0.5 ml. The leg was then elevated and massaged. The surgical site in the inguinal region was then explored, and within 5 min of injection, blue lymph vessels and leaks were noted. See Figs. 1, 2, and 3 for intraoperative photographs. All these were ligated with titanium clips. The wound was then either closed in layers with or without drainage (operator dependent) or closed with vacuum-assisted closure (VAC) because of contamination of the surgical site. Drains were removed when there was a production of less than 50 ml within 24 h.

## Results

In total, lymph vessel ligation was performed ten times in the inguinal region in a total of eight patients as one case was a bilateral case and one case was treated twice. Four were male, and the median age at surgery was 70 years (range 44–78 years). The median number of days from original surgery to time of lymph vessel ligature was 32 days (range 15–144). The patients were all treated in the periods 2007 and 2015, and the minimum follow-up time was 4 months. The patient characteristics are summarized in Table 1.

**Table 1:** Overview of treated patients

Patient number	Gender	Age at surgery	Cause	Days until ligation	Side	Number of lymph vessels ligated	Drain/VAC	Days until drain/VAC removal	Postoperative need for puncture
1	Female	44	Melanoma	32	Left	2	Drain	4	No^a^
2	Female	78	Lymphoma	84	Left	4	None	–	No
3	Female	62	Melanoma	32	Right	5	Drain	21	No
4	Male	60	Penile SCC	39	Left	3	VAC	17	No
5	Male	70	Melanoma	26	Bilateral	3/2	Drain	1/5	Yes/yes
6	Female	70	Melanoma	27	Right	2	Drain	3	Yes
7	Male	76	Melanoma	15	Right	3	VAC	5	No
8	Female	72	Ovarian cancer	144	Right	1	None	–	No

*VAC* vacuum-assisted closure

^a^Postoperatively developed lymphatic malformation, which was surgically removed. After this procedure, lymphocele developed again which in the end was treated with a renewed vessel ligation procedure

Preoperatively, a median of three blue leaking lymph vessels were found and ligated (range 1–5 vessels). In six cases, the wound was closed routinely in layers, and in the remaining two cases, VAC was used due to the contamination of the surgical site. In cases with direct suture, drains were placed based on the preference of the surgeon. Drains were removed when the daily production was less than 50 ml.

For two patients including the bilateral case, there was a need for postoperative fluid drainage which ranged from 13 to 37 days to the last puncture date. Another patient with an immediate good response to vessel ligation was suspected of having recurrence of melanoma 3 months after vessel ligation. PET-CT revealed that the inguinal bulge was a lymphatic malformation which was removed surgically. Lymphocele formation followed the removal of the lymphatic malformation and was treated conservatively for 5 months without effect. The lymph vessel ligation procedure was repeated with an immediate positive response again. In the remaining five patients, there was a complete immediate response without recurrence. Once the lymphocele formation had ceased, no one had recurrence in the follow-up period.

No other postoperative complications were noted in any cases.

## Discussion

In total, we have performed this procedure in eight patients with a total of ten inguinal regions. In two patients (three regions), further puncture of the surgical site was necessary up to about 1 month after surgery. In these two patients, the mean duration of the lymphocele was just less than 1 month and one can wonder if these cases would have regressed spontaneously in the same time frame without surgery. In the remaining cases, there was no need for further puncture after the procedure. Some of the effect attributed to this procedure might also be contributed by the postoperative immobilization, which is often recommended to minimize fluid accumulation.

Previous literature regarding ligature of lymph vessels is very sparse, but previous case reports and series all show improvement postoperatively in all cases [3–5]. In our series, we show that the need for puncture does not necessarily end right after surgery and one can expect that in some cases, there will be a need for further drainage in the first few weeks postoperatively. The same case reports have injected the Patent Blue V in the food distally in the interdigital spaces; however, we have opted to inject the dye at the level of the ankle and in all cases, we could identify blue lymph vessels in the inguinal area.

We did not experience any postoperative complications with this procedure besides one patient that developed a lymphatic malformation. This was surgically removed, but resulted in the recurrence of lymphocele which was then treated again with immediate effect. It is a very easy, simple, and quick procedure that has the potential to benefit patients where other options are sparse. In six out of eight patients, this procedure led to an immediate stop to the lymphocele formation.

It is interesting to note that the patients that did not have an immediate stop of their lymphocele had drains placed which were removed within a few days after surgery due to low production in the drains. Patients without drains or with VAC closure all had immediate effect of the procedure. The patient material however is very small, and nothing can be concluded on whether to use drains or not.

One could also hypothesize that this procedure could be used while performing the actual lymphadenectomy to minimize the risk of later lymphocele formation. This is however just a speculation and would need to be examined in a properly designed clinical trial.

## Conclusions

Ligation of lymph vessels can be an effective treatment modality for recurrent inguinal lymphoceles following inguinal lymphadenectomy where the lymphocele formation is not decreasing or the fluid accumulation is just too dramatic to warrant a conservative approach. The treatment is quick and easy to perform but should be reserved for the unmanageable cases to avoid overtreating patients as the condition is usually self-limiting. In the severe cases, lymph vessel ligation can be recommended but the evidence for this treatment is still only on a case series basis.
